# Numerical Investigation of Crack Suppression Strategies in Ultra-Thin Glass Substrates for Advanced Packaging

**DOI:** 10.3390/mi16111256

**Published:** 2025-11-01

**Authors:** Xuan-Bach Le, Kee-Youn Yoo, Sung-Hoon Choa

**Affiliations:** 1 Energy & Environment Research Institute, Seoul National University of Science and Technology, Seoul 01811, Republic of Korea; lexuanbach@seoultech.ac.kr; 2 Department of Chemical and Biomolecular Engineering, Seoul National University of Science and Technology, Seoul 01811, Republic of Korea; kyyoo@seoultech.ac.kr

**Keywords:** glass substrates, edge crack, energy release rate, redistribution layer (RDL), finite element analysis (FEA)

## Abstract

The mechanical reliability of glass substrates is a key challenge for their adoption in advanced semiconductor packaging. This study employs finite element analysis to systematically evaluate the risk of edge crack propagation in large glass panels during redistribution layer (RDL) fabrication. The influence of critical factors—including crack location, number of RDLs, glass material and thickness, dielectric ABF properties, Cu content, and edge clearance—was examined. Results revealed that top-edge crack near the RDL/glass interface pose the highest failure risk due to elevated peeling stress and increased energy release rate (ERR). The risk of propagation intensifies with more RDLs and thinner glass, while high CTE (coefficients of thermal expansion) glasses such as D263, Gorilla, and ceramic glass markedly suppress crack growth compared with borofloat 33 and fused silica. Among ABF dielectrics, GZ-41 demonstrated superior crack resistance owing to its low CTE and moderate stiffness. Although higher Cu content slightly reduced ERR, its effect remained limited. Edge clearance strongly affects reliability, with ≥300 µm providing effective suppression of crack propagation. These findings provide quantitative design guidelines for glass interposer structures, emphasizing the optimization of dielectric material selection, glass substrate and thickness, and layout constraints such as edge clearance. The proposed methodology and results will contribute to establishing reliable strategies for deploying ultra-thin glass panels in advanced semiconductor packaging.

## 1. Introduction

The continuous advancement of semiconductor device performance has driven innovations in microelectronic packaging technologies. In particular, advanced packaging architectures such as 2.5D and 3D integration play a critical role in enabling applications including high-performance computing, artificial intelligence (AI), and autonomous vehicles [1,2,3]. These applications demand substrates with high-density interconnects, excellent electrical performance, robust mechanical reliability, and cost-effectiveness. Among the various substrate candidates, glass has emerged as a highly promising material due to its excellent electrical properties, capability for large-area fabrication leading to potential cost savings [4,5], tailorable coefficient of thermal expansion (CTE) and smooth surface [6,7].

Nevertheless, several challenges limit the practical use of glass substrates, including limited thermal dissipation, poor adhesion, and high susceptibility to cracking [8,9]. Manufacturing processes such as through-glass via (TGV) formation, chemical mechanical polishing (CMP), and dicing or singulation of the glass panel often introduce micro-defects into the glass [10,11]. In particular, crack initiation and propagation in glass substrates remain major obstacles to their commercialization. Despite the critical importance of reliability issues such as crack formation, systematic investigations into these issues still remain insufficient. Lee et al. developed a simulation methodology to accurately predict and control the warpage of glass substrate in fan-out panel-level packaging (FO-PLP) architectures [12,13,14,15,16,17]. However, their work mainly addressed warpage induced by residual stresses from RDL fabrication process and did not investigate the risk of crack propagation. Wei et al. first reported the so-called “SeWaRe” failure phenomenon, in which glass substrate edges peel back like the pages of a book during the dicing process [11]. McCann et al. [10,18,19,20,21] investigated the crack propagation mechanism in glass panels during the thermal cycling tests, emphasizing critical factors including total RDL build-up thickness, edge defects during dicing, and environmental moisture effects. They proposed solutions such as edge coating, pull-back, and optimized dicing methods to mitigate crack growth. Recently, Sunohara et al. [22] developed a glass core build-up substrate and experimentally demonstrated that SeWaRe cracking occurs at the edge of the glass substrate near the glass/RDL interface.

A distinctive feature of crack failure in glass substrates is the progressive propagation of the crack during the fabrication process. During redistribution layer (RDL) fabrication of the glass substrate, copper (Cu) and dielectric layers such as Ajinomoto build-up film (ABF) are deposited at elevated temperatures [18,23]. Upon cooling to room temperature, tensile stresses develop due to CTE mismatch between the RDLs and the glass substrate. These stresses can act on pre-existing micro-defects, promoting crack initiation and Mode I fracture, which can lead to catastrophic substrate failure. As the number of RDL and ABF layers increases, the cracks keep propagating due to the accumulation of thermal strain energy induced by CTE mismatch. As advanced packaging technology advances toward ultra-thin glass substrates (≈100 µm) and increased RDL stacking [24,25], the risk of glass cracking becomes even more pronounced. Therefore, it is very important to minimize the crack propagation during fabrication process.

Despite its significance, a comprehensive understanding of the influence of RDL architecture, glass properties (material and thickness), dielectric materials, and edge defect severity on crack propagation in large-area glass panels is still lacking. To address this gap, the present study systematically investigated the risks of edge crack propagation throughout the RDL fabrication process and suggested the crack suppression strategies in glass substrates. Finite element simulations were employed to examine critical parameters, including glass thickness, ABF dielectric properties, Cu volume fraction, and edge clearance, in large-area glass panels (510 mm × 515 mm). These results will provide design strategies to mitigate glass cracking and facilitate the use of ultra-thin glass substrate in advanced semiconductor packaging.

## 2. Simulation Modeling and Boundary Condition

### 2.1. Equivalent RDL Architecture and Material Properties

In this study, a finite element analysis (FEA) approach was used to assess the risk of crack propagation during the RDL fabrication process. A glass panel with dimensions of 510 mm × 515 mm and thicknesses ranging from 100 μm to 500 μm was used. Four RDLs were sequentially fabricated on each side of the glass substrate by repeating polymer (ABF) lamination, Cu seed deposition, photolithography, and electroplating. The same build-up sequence was applied simultaneously on both sides. Direct meshing of full-panel RDL structures was computationally challenging due to their intricate patterning and multi-scale features. Therefore, an equivalent material approach was employed, in which the multilayer RDL stack (Cu/ABF) was homogenized into a single effective layer using established simulation-based material evaluation methodologies [13,23].

**Figure 1:**
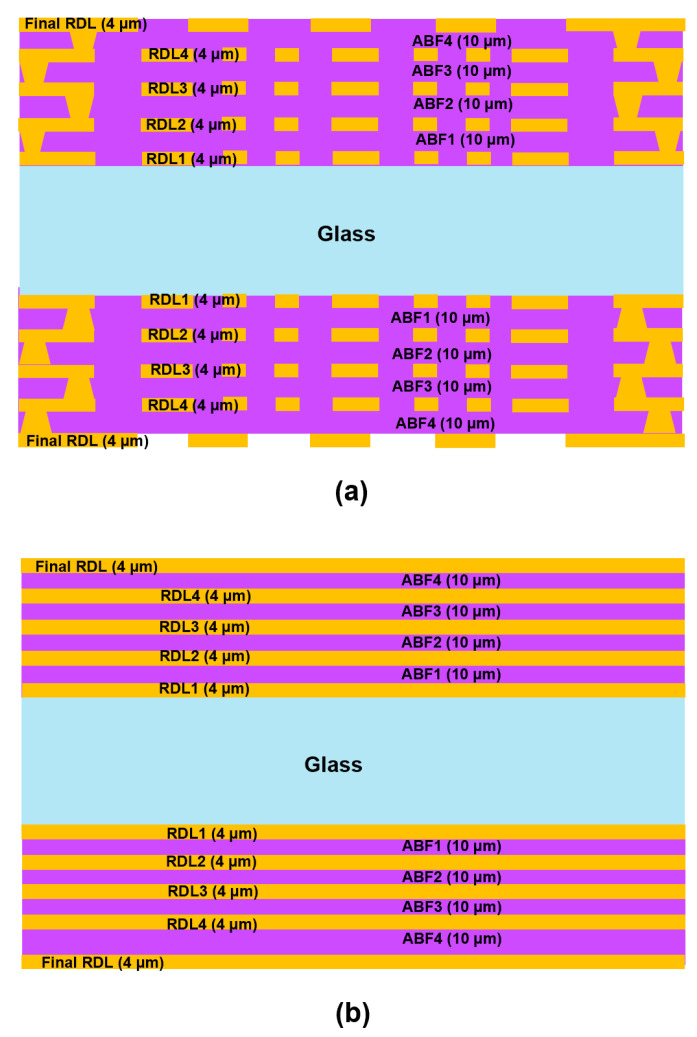
(**a**) Actual RDL architectures; (**b**) Simplified RDL architectures.

Figure 1 illustrates both the actual and simplified stacking RDL architectures. In the FEA model, the actual RDL structure composed of alternating Cu and ABF polymer layers was replaced with a single equivalent layer as shown in Figure 1b, whose mechanical properties were determined using a representative volume element (RVE) approach with simulation-based material evaluation. Copper and ABF polymer were assumed to be uniformly distributed within the RDL structure. For this study, the Cu volume fraction was arbitrarily set to 50%, although it can be modified to accommodate various package designs or fabrication conditions.

**Figure 2:**
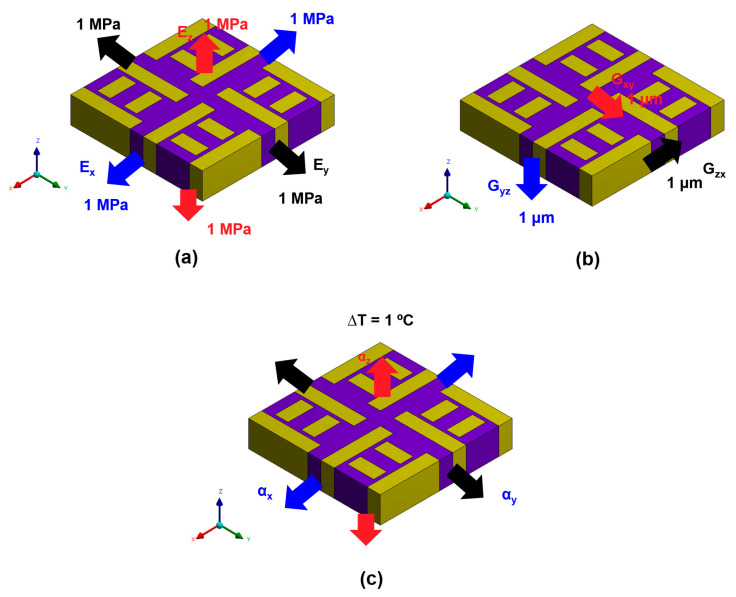
Boundary conditions of the equivalent material model: (**a**) Uniaxial tensile loading; (**b**) Shear loading; (**c**) Thermal loading.

The equivalent material properties, including Young’s modulus, shear modulus, Poisson’s ratio, and CTE were obtained by applying simulated tensile, shear, and thermal expansion load cases to a simplified RVE model, as shown in Figure 2. The ABF layers between adjacent RDLs contain several Cu-filled vias. However, these vias were neglected in the FEA due to their low Cu content, and the ABF layers were modeled solely as ABF material. For the detailed build-up structure, all RDLs (RDL1–RDL4 and the final RDL) had a thickness of 4 µm, and all ABF dielectric layers had a thickness of 10 µm.

The stress-free temperature of the equivalent RDL was determined by matching the simplified model against the actual multilayer RDL. Specifically, the reference temperature of the equivalent layer was chosen such that the thermal warpage predicted by the simplified RVE model matched that of the detailed RDL, following the procedure described in the modified Timoshenko bi-material theory [23]. The validity of this equivalent RVE approach was extensively and experimentally validated in the previous studies by Lee et al. [12,13,14,15], in which the thermal warpage predicted by the RVE model exhibited excellent agreement with the measured warpage of the detailed RDL structure (error < 5%). Therefore, in this study, the RVE method was directly adopted to represent the RDL. The stress-free temperature of the equivalent RDL can be expressed by the following equation, where X, Y, and Z are constants determined by the geometric configuration and Young’s modulus of the constituent materials”.

(1) $$T_{stress-free}=\frac{V_{ABF}^{2}\times X\times \left(\alpha_{glass}-\alpha_{ABF}\right)}{Y\times \left(\alpha_{glass}-\alpha_{eq}\right)}\times \left(T_{ABF}-T_{Room}\right)\;\;+\frac{V_{Cu}^{2}\times X\times \left(\alpha_{glass}-\alpha_{Cu}\right)}{Z\times \left(\alpha_{glass}-\alpha_{eq}\right)}\times \left(T_{ABF}-T_{Room}\right)+T_{Room}$$

(2) $$X=\left(3{\left(1+\frac{t_{1}}{t_{2}}\right)}^{2}+\left(1+\frac{t_{1}E_{eq}}{t_{2}E_{glass}}\right)\left(\frac{t_{1}^{2}}{t_{2}^{2}}+\frac{t_{2}pE_{glass}}{t_{1}E_{eq}}\right)\right)$$

(3) $$Y=\left(3{\left(1+\frac{t_{1}}{t_{2}}\right)}^{2}+\left(1+\frac{t_{1}E_{ABF}}{t_{2}E_{glass}}\right)\left(\frac{t_{1}^{2}}{t_{2}^{2}}+\frac{t_{2}E_{glass}}{t_{1}E_{ABF}}\right)\right)$$

(4) $$Z=\left(3{\left(1+\frac{t_{1}}{t_{2}}\right)}^{2}+\left(1+\frac{t_{1}E_{Cu}}{t_{2}E_{glass}}\right)\left(\frac{t_{1}^{2}}{t_{2}^{2}}+\frac{t_{2}E_{glass}}{t_{1}E_{Cu}}\right)\right)$$

where V_Cu_ and V_ABF_ are the volume fractions of Cu and ABF, respectively. α_Cu_, α_glass_ and αep are the CTE of Cu, glass, and equivalent RDL, respectively. T_Cu_, T_ABF_ are the stress-free temperature of Cu and ABF, respectively. The thickness of the RDL and the glass are t_1_ and t_2_, respectively. E_Cu_, E_ABF_, E_glass_ and E_eq_ are the Young’s modulus of Cu, ABF, Glass, and equivalent RDL, respectively.

Four ABF materials widely used in advanced packaging were selected for this study: GX-92, GL-102, GZ-41, and GZ-22. Among them, GX-92 is the most widely used in industry and was therefore chosen as the reference ABF material in this study [20,26,27,28]. In addition, five different glass substrates were investigated: fused silica, borofloat 33 (a borosilicate glass, Schott), D263 (an alkali-borosilicate glass, Schott), Gorilla glass, and ceramic glass. For the simulations, the reference condition in this study was selected as borofloat 33 glass with a thickness of 210 μm combined with ABF GX-92 material. The detailed material properties used in the simulations are listed in Table 1 and Table 2.

### 2.2. Mesh Modeling and Edge Crack Definition

**Figure 3:**
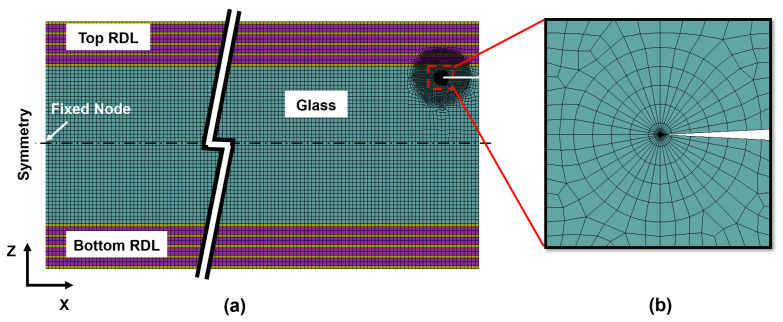
Structure and mesh modeling of the standard model with initial horizontal edge crack: (**a**) mesh modeling; (**b**) mesh of crack tip.

A two-dimensional (2D) plane strain FEA model was created using ANSYS Workbench 2022 software. Symmetry conditions were applied along the left edge of the structure as shown in Figure 3a. To simulate process-induced defects, an initial horizontal crack was introduced at the free edge on the right side of the glass panel.

**Figure 4:**
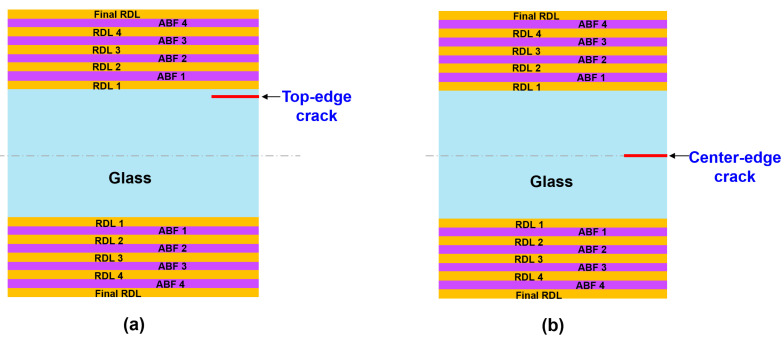
Schematic illustration of initial crack locations: (**a**) Top-edge crack; (**b**) Center-edge crack.

While cracks may initiate at various depths along the glass edge, this study focused on two critical and commonly observed failure sites: (1) a top-edge crack near the glass/RDL interface, positioned 15 μm from the glass/RDL interface and (2) a center-depth edge crack (or center-edge crack), reflecting edge defects typically introduced during dicing [11,21,22]. These two crack locations are schematically illustrated in Figure 4. Initial crack lengths ranging from 2 μm to 50 μm were considered, reflecting the typical defect sizes introduced during TGV fabrication and dicing processes.

A typical mesh near the crack tip is shown in Figure 3b. A refined mesh was generated around the crack tip to accurately resolve the stress singularity. The cracks were modeled as propagating inward from the edge of a glass panel, with propagation driven by tensile stresses (Mode I). The J-integral (contour integral) method was used to compute the energy release rate (ERR) at the crack tip using the following formula [30]:

(5) $$J\;=\int_{\Gamma }wdy-\;T\frac{\partial u}{\partial x}ds$$

where Γ is a counterclockwise path enclosing the crack tip, W is the strain energy density, x and y are the in-plane directions, *T* is the traction vector, u is the displacement vector, ds is a length increment along the contour Γ. For linear, brittle, and isotropic materials such as glass, the J-integral is equivalent to the strain energy release rate (ERR). Crack propagation occurs when the ERR exceeds the critical ERR value (Gc), which is a material property representing the fracture toughness of the material. To improve accuracy, multiple contours were generated around the crack tip, and the converged J-integral value within the contours was selected as the final result. In addition, the effect of moisture on reducing the fracture toughness of glass was considered, since water is commonly present during dicing and cleaning processes. Previous studies have reported that the critical ERR of borosilicate glass decreases from 8 J/m^2^ in air to 1.98 J/m^2^ in water [10,18]. Accordingly, in this study, 1.98 J/m^2^ was taken as the critical threshold for crack propagation, and the calculated ERR was compared with this value to assess the risk of crack propagation.

### 2.3. Process-Oriented Simulation of RDL Fabrication

**Figure 5:**
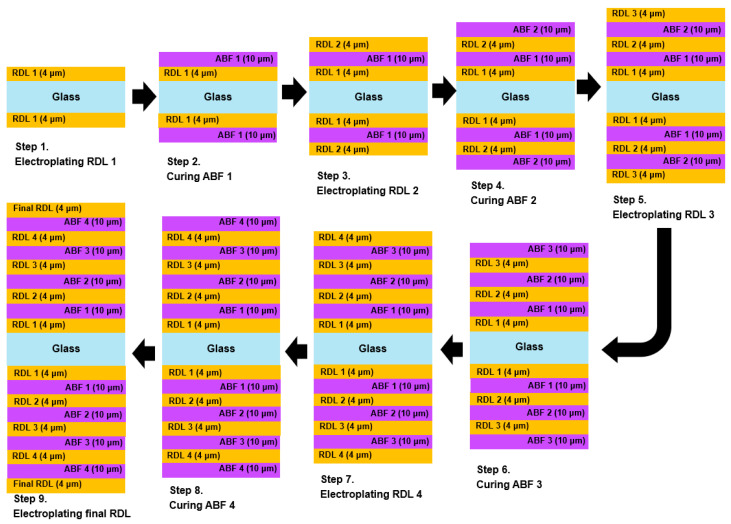
Process-oriented simulation flowchart of the RDL fabrication process.

After defining the equivalent material properties and introducing the initial crack, a process-oriented simulation was implemented to replicate the panel-level RDL fabrication sequence, as shown in Figure 5. In each step, RDL and ABF layers were built simultaneously on both sides of the glass panel, in accordance with the typical panel-level manufacturing sequence [18,31,32]. At each step, the panel was heated to the designated curing temperature for the ABF layer or the processing temperature for RDL, after which the layer was deposited. The glass panel was then cooled to room temperature before proceeding to the next step. This procedure was iteratively repeated until the entire multilayer RDL/ABF structure was completed.

In the simulation, the element birth-death technique was used to simulate the actual fabrication sequence of the RDL. At the beginning of the process simulation, only the glass substrate was present. Therefore, the simulation started with “birth” the glass panel at room temperature (25 °C), and “death” all other layers or materials. Subsequently, each RDL and dielectric layer was sequentially “birthed” at its respective process (curing or plating) temperature, which was defined as the stress-free condition in the thermo-mechanical analysis. Specifically, the temperature was increased from room temperature to the stress-free temperature of the first RDL (117 °C), at which point RDL1 was activated (element birth) on both surfaces of the glass panel without residual stress. The structure was then cooled to room temperature. Subsequently, the temperature was increased to the curing temperature of the first ABF layer (180 °C), at which point ABF1 was activated (element birth) on both surfaces of the glass panel without residual stress, followed by cooling to room temperature. This thermal cycle was systematically repeated for each successive RDL and ABF layer, applied in parallel on both surfaces of the glass panel, until the final RDL was completed. The heating and cooling rates were both set to 5 °C/min, and no soaking period was included.

## 3. Results and Discussion

### 3.1. Effect of Crack Location and the Number of RDLs on the Risk of Crack Propagation

**Figure 6:**
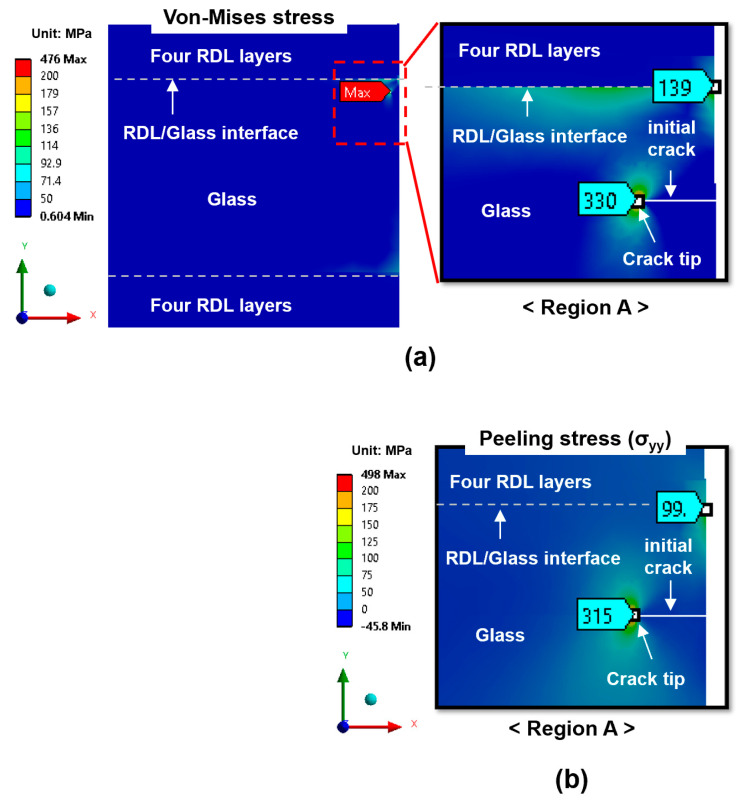
Stress distribution maps for the top-edge crack after four-layer RDL fabrication: (**a**) von-Mises stress; (**b**) peeling stress (σ_yy_).

To investigate the mechanisms responsible for crack propagation in the glass substrate, the stress distribution was analyzed to identify critical regions. Figure 6 shows the stress distribution for the case of a top-edge crack after the fabrication of final RDLs with an initial crack length of 10 µm. The highest von Mises stress, about 330 MPa, was concentrated at the crack tip (region A in Figure 6a), indicating that this location is the most critical site for failure. To further evaluate the driving force for crack propagation, the peeling stress was also analyzed. To further assess the driving force for propagation, the peeling stress (σ_yy_), defined as the normal stress acting perpendicular to the RDL/glass interface and responsible for Mode I crack opening, was also evaluated. As shown in Figure 4b, the maximum peeling stress of 315 MPa was also found at the crack tip.

**Figure 7:**
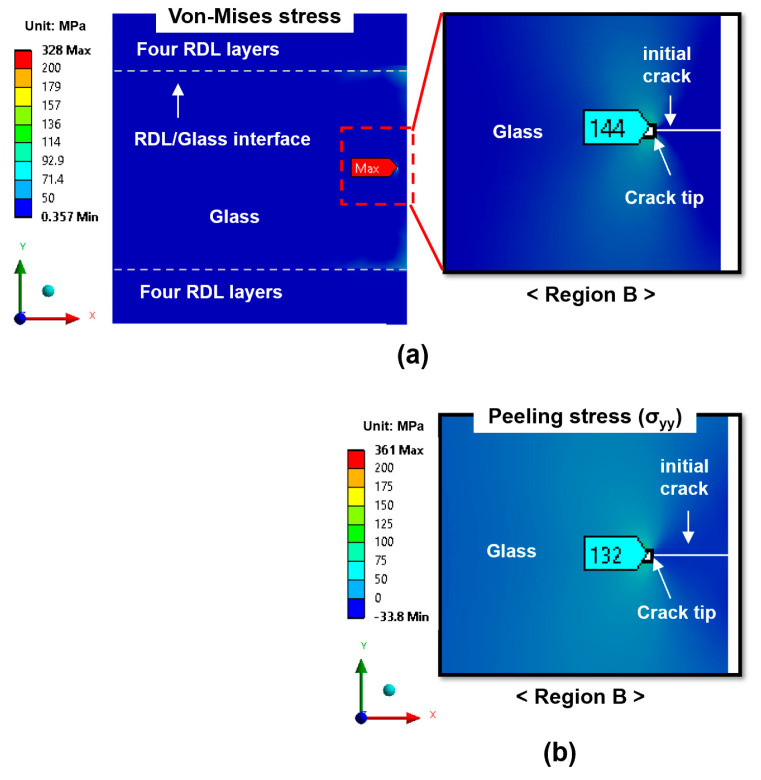
Stress distribution maps for the center-edge crack after four-layer RDL fabrication: (**a**) von-Mises stress; (**b**) peeling stress (σ_yy_).

Besides the crack tip, the RDL/glass interface near the side edge of the glass panel represents another critical region of concern, as it may serve as a potential initiation site for delamination between the glass substrate and RDL. However, at this interface, the von-Mises and peeling stresses were 139 MPa and 99 MPa, respectively—approximately 2.5 times lower than those at the crack tip. Thus, glass cracking is considered as the primary reliability concern in this study. As shown in Figure 7, the von-Mises stress and peeling stress for the center-edge crack are 144 MPa and 132 MPa, respectively, which are substantially lower than those for the top-edge crack. This suggests that the risk of center-edge crack propagation is considerably lower.

**Figure 8:**
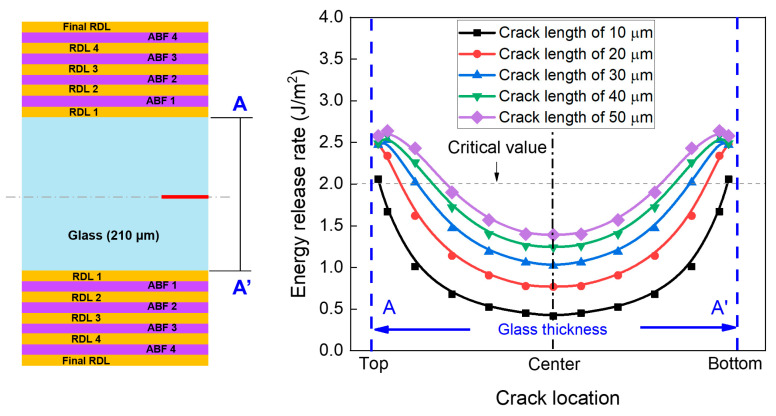
Effect of crack location on the risk of crack propagation after the final RDL fabrication.

To further clarify the influence of crack location on the risk of crack propagation, the effect of crack location on ERR values was investigated. Figure 8 shows the effect of crack location and initial crack length on the ERR values after the final RDL fabrication. The crack was introduced at different positions along the glass thickness direction (A-A′), with the crack length ranging from 10 µm to 50 µm. The ERR is lowest when the crack is located at the center-edge of the glass and increases significantly as the crack approaches closer to the RDL/glass interface. This trend is consistent with the stress distribution in Figure 6 and Figure 7, where the top-edge crack exhibits much higher stress than the center-edge crack. This behavior is attributed to thermo-mechanical stresses induced by the CTE mismatch between the RDL/ABF layer and the glass substrate during cooling. The cracks near the interface are subjected to higher peeling stress, resulting in higher ERR values, whereas cracks at the center edge experience lower stresses and correspondingly lower ERR values.

These results indicate that the top-edge crack is the most critical; therefore, the subsequent analysis in this study focuses on this case. The predicted failure location is consistent with the experimental observations reported by Sunohara et al. [22], where top-edge cracking near the RDL/glass interface was identified as the dominant failure mode in glass panels. Moreover, the failure induced by top-edge cracks may be further accelerated by the CMP process.

**Figure 9:**
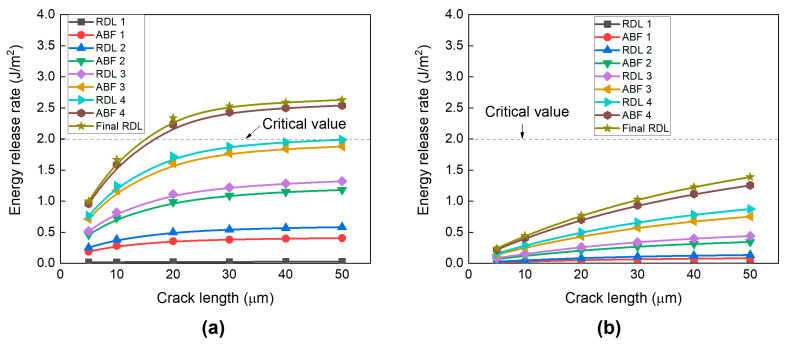
Effect of number of RDL on risk of crack propagation as a function of crack length: (**a**) Top-edge crack; (**b**) Center-edge crack.

Next, the effect of the number of RDLs on risk of crack propagation was analyzed by comparing the calculated ERR values with the critical ERR of borosilicate glass (2 J/m^2^). Figure 9 presents the variation in ERR with crack length for (a) top-edge crack and (b) center-edge crack under different numbers of RDLs. The ERR value increases linearly as the number of RDL and ABF layers increases owing to the accumulation of thermal strain energy induced by CTE mismatch, thereby increasing the risk of crack propagation. As expected, increasing the crack length also results in higher ERR values for both crack types. However, the effect of crack length on ERR values differed between the two crack locations. For the top-edge crack (as shown in Figure 9a), the ERR increases linearly with crack length, but approached a plateau beyond 30 μm. The risk of crack propagation becomes significant at three RDLs and exceeds the critical threshold with four layers, indicating a high risk of glass cracking in the four-RDL structure.

In contrast, for center-edge cracks (as shown in Figure 9b), the ERR continues to increase linearly without saturation up to crack length of 50 μm. Overall, the ERR values remain lower than those for top-edge cracks and do not exceed the critical threshold, even with four RDLs.

### 3.2. Effect of Glass Thickness and Glass Materials on Risk of Crack Propagation

**Figure 10:**
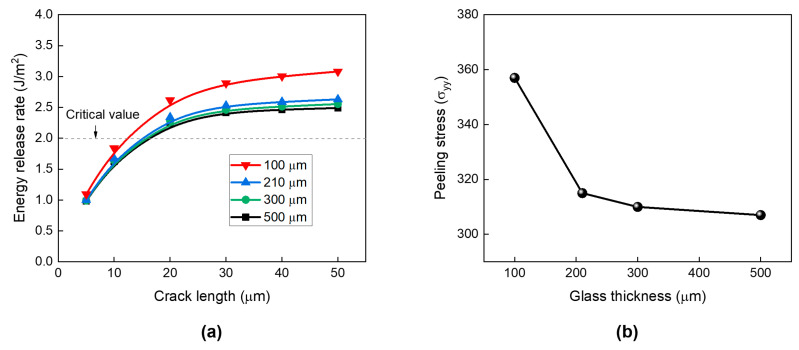
Effect of glass thickness on (**a**) the risk of crack propagation for a top-edge crack for different crack lengths; (**b**) peeling stress (σ_yy_) around the top-edge crack tip.

The risk of crack propagation can also be affected by the thickness of the glass substrate. Figure 10a presents the calculated ERR values of borofloat 33 glass with thicknesses ranging from 100 µm to 500 µm after the final RDL fabrication. The ERR increases only slightly (≈5%) as the thickness decreases from 500 µm to 210 µm, indicating a minor effect in this range. However, when the glass thickness is further reduced to 100 µm, the ERR increases significantly by nearly 30%, making the glass highly susceptible to cracking.

This tendency agrees well with the previous experimental study [32], where the 100 µm glass substrate showed slightly higher stress compared with the 300 µm glass substrate. This distinct behavior of the 100 µm glass is related to the rigidity of the glass substrate. According to the Kirchhoff–Love plate theory, the bending stiffness D is proportional to t^3^, where t is the glass substrate thickness [33]. When the thickness decreases from 500 µm to 100 µm, the flexural rigidity is drastically reduced, leading to much higher tensile (peeling) stress at the glass edge under thermal loading. Consequently, the 100 µm-thick glass substrate exhibits a pronounced edge-stress concentration, substantially increasing the likelihood of crack initiation. Figure 10b shows the peeling stress around the crack tip of the top-edge crack for different glass thicknesses. The peeling stress increases as the glass becomes thinner, and at a thickness of 100 µm, it rises sharply due to the severe loss of structural rigidity.

**Figure 11:**
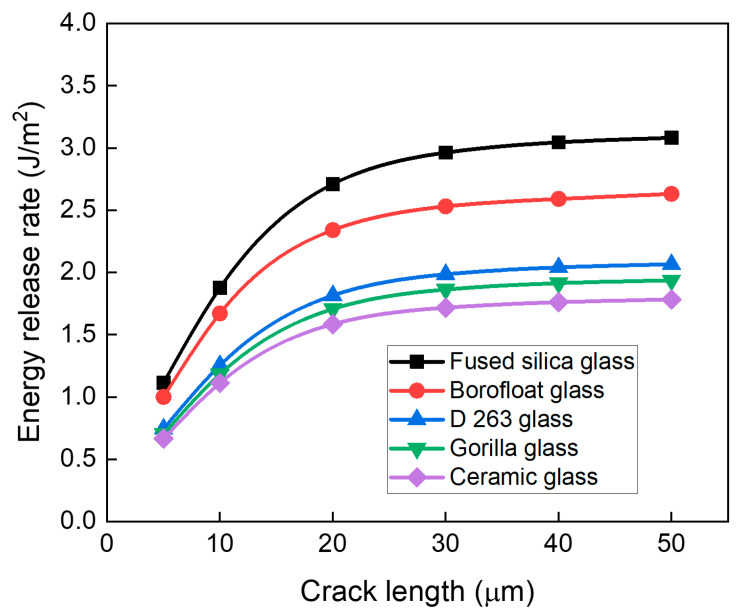
Effect of glass materials on the risk of crack propagation for a top-edge crack for different crack lengths.

In addition to thickness, the choice of glass material also plays a crucial role in determining crack resistance. Since each glass exhibits a unique critical energy release rate (Gc), crack propagation occurs once the ERR exceeds this threshold. Therefore, it is important to examine the effect of crack length on ERR for different glass materials. Five different glass materials were investigated: fused silica, borofloat 33, D263, Gorilla glass, and ceramic glass. Figure 11 presents the ERR values of five glass materials with a thickness of 210 µm for the top-edge crack after the final RDL process. Fused silica glass shows the highest ERR value for all crack lengths, while ceramic glass shows the lowest ERR value. Notably, D263, Gorilla glass, and ceramic glass have significantly lower ERR values compared with borofloat 33 and fused silica, indicating their effectiveness in suppressing crack propagation.

The CTE values of these glasses are as follows: fused silica (0.57 ppm/°C), borofloat 33 (3.25 ppm/°C), D263 (7.2 ppm/°C), Gorilla glass (8.14 ppm/°C), and ceramic glass (9.3 ppm/°C), whereas copper of RDL has a much higher CTE of 17.6 ppm/°C. As a result, glass substrate with higher CTE tend to show lower ERR values due to the reduced CTE mismatch with copper. Besides CTE, Young’s modulus of glass also affects ERR. However, the values for the five investigated glasses vary only slightly, from 64 to 72.9 GPa (Table 3). This narrow range exerts only a minor effect on ERR compared with the large differences in CTE. Thus, variation in glass CTE is the primary factor governing crack propagation.

**Figure 12:**
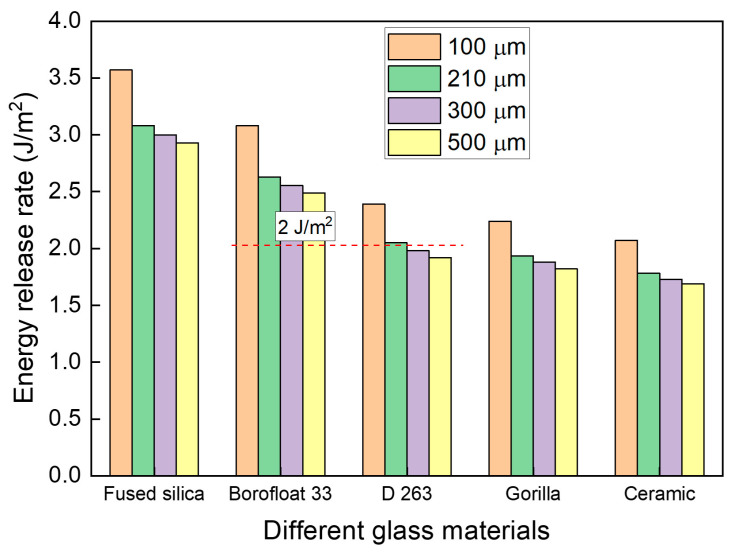
Effect of glass thickness on the ERR of each glass material at a crack length of 50 µm.

Figure 12 illustrates the summery of the influence of glass thickness and glass material on ERR at a crack length of 50 µm. Notably, for D263 glass, a thickness greater than 300 µm effectively suppresses crack propagation, even at an initial crack length of 50 µm. In general, the ERR increases as the glass thickness decreases for all glass materials, reflecting the lower resistance of thinner glass substrates to edge crack propagation. However, the effect of glass thickness is relatively smaller than that of glass material.

### 3.3. Effect of ABF Materials and Cu Content on Risk of Crack Propagation

Since edge cracks are mainly driven by thermal-mechanical stress, the material properties of the ABF dielectric layer also play an important role in determining the risk of crack propagation. ABF materials typically possess a CTE in the range of 22~46 ppm/°C [26,27,28,29], which is considerably higher than that of Cu (17.6 ppm/°C) and glass (0.57~9.3 ppm/°C) [9]. In contrast, the Young’s modulus of ABF ranges from 4 to 13 GPa [26], which is much lower than that of Cu (91.7 GPa) and glass (64~72.9 GPa) [9]. Therefore, both CTE and Young’s modulus must be considered in ABF material selection to ensure the reliability of glass panel. In this study, we investigated four ABF materials (GL-102, GX-92, GZ-22 and GZ-41) widely used in industry, and the material properties are summarized in Table 4. Among them, GL-102 has the lowest CTE and the highest Young’s modulus. GX-92 has the highest CTE and the lowest Young’s modulus. GZ-22 has the moderate CTE and the relatively low Young’s modulus. GZ-41 has the same CTE as GL-102 and a moderate Young’s modulus.

**Figure 13:**
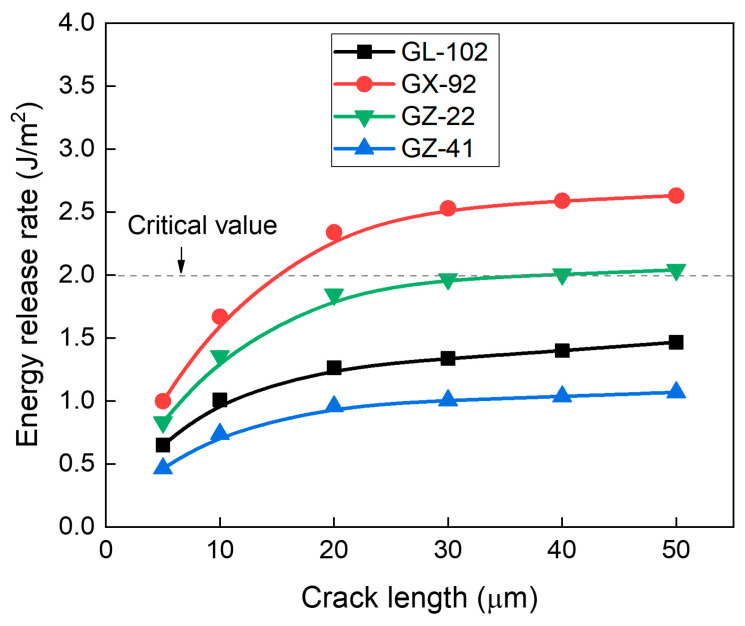
Effect of ABF materials on the risk of top-edge crack propagation as a function of crack length.

Figure 13 demonstrates that GL-102 and GZ-41 possess substantially lower ERR values compared with the other ABF materials. Importantly, their ERR values remain below the critical threshold for crack propagation, indicating a substantially reduced fracture risk. Among the four ABF materials investigated, GZ-41 exhibits the lowest ERR value, mainly due to its low CTE combined with a moderate Young’s modulus. These results suggest that an optimal approach to suppressing crack propagation is the use of ABF materials combining a low CTE with a moderate Young’s modulus.

**Figure 14:**
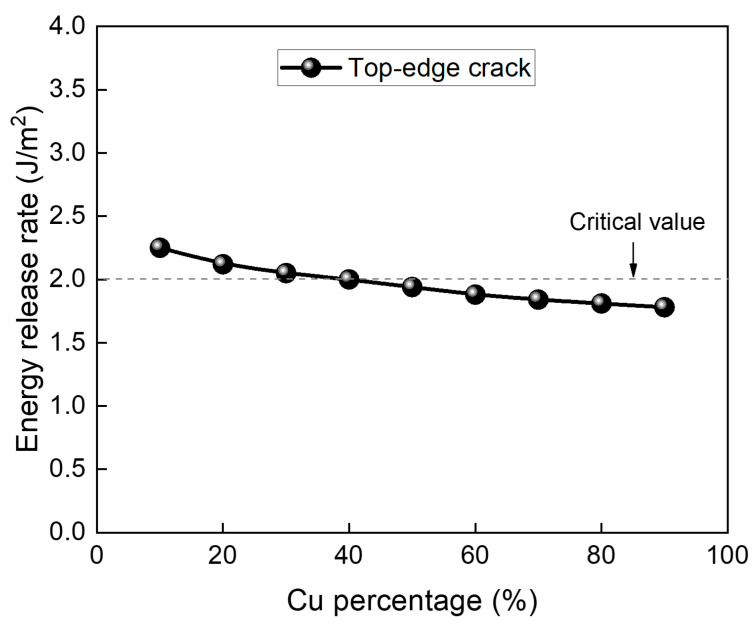
Effect of Cu percentage on top-edge crack propagation risk when using ABF GX-92 for a 15 µm crack length.

In addition to ABF properties, the Cu content in the RDL also impacts the thermo-mechanical stress in the glass substrate. As summarized in Table 5, increasing the Cu percentage increases the effective Young’s modulus of the equivalent RDL, thereby increasing the thermo-mechanical stress, while simultaneously reducing the CTE and stress-free temperature, which lowers thermo-mechanical stress. The relationship between Cu content and the stress-free temperature of the equivalent RDL is governed by Equations (1)–(4). Figure 14 shows the effect of Cu percentage on top-edge crack propagation risk when using ABF GX-92 with a crack length of 15 µm. Due to these competing effects, increasing Cu content from 10% to 90% reduces the ERR by only ~20%. This suggests that at low Cu percentages, ABF material dominates the thermo-mechanical stress behavior, producing higher thermo-mechanical stress and slightly increasing the crack propagation risk.

### 3.4. Effect of Edge-Clearance on Risk of Crack Propagation in Glass Panel

**Figure 15:**
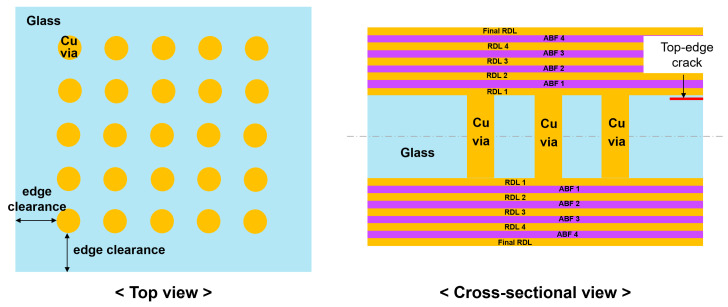
Schematic drawings of edge clearance with a top-edge crack in glass interposer structure.

Edge clearance, defined as the distance from the outermost Cu via to the glass edge as shown in Figure 15, is also an important factor that affects the risk of crack propagation. In industries, edge clearance was used to prevent micro-defects generated during dicing or post-processing [19,32]. If Cu vias are placed too close to the glass edge, edge defects or thermo-mechanical stresses can easily trigger crack initiation and propagation. On the while, increasing edge clearance leaves unused space lowering glass panel efficiency and reduced TGV density. In this study, the effect of edge clearance on the risk of crack propagation was analyzed using a typical glass interposer structure with Cu vias (via diameter of 50 μm, pitch/diameter ratio of 2), as shown in Figure 15. We assumed that the top-edge crack existed in this glass interposer structure.

**Figure 16:**
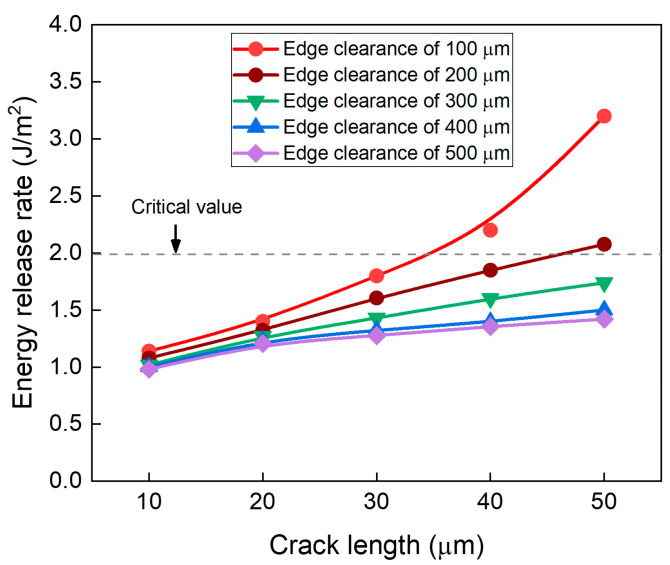
Effect of edge clearance on the risk of crack propagation as a function of crack length.

Figure 16 shows the ERR results as a function of crack length for different edge clearances for 500-μm-thick borofloat 33 glass using ABF GL-102. As the edge clearance increases, the ERR decreases, clearly indicating that larger clearances reduce the risk of edge crack propagation. For an edge clearance of 100 µm, the ERR rapidly exceeds the critical threshold of cracking beyond a crack length of 40 µm, demonstrating that this margin is insufficient to prevent crack propagation. With 200 µm clearance, the ERR remains close to the threshold, suggesting that greater than 200 µm is required to prevent propagation. On the whole, edge clearances of 300 µm or greater maintain ERR values well below the threshold, ensuring robust resistance during RDL fabrication.

## 4. Conclusions

This work presents a comprehensive numerical study on the mechanisms and prevention of crack propagation in glass panels during RDL fabrication, focusing on critical parameters such as glass thickness, glass materials, ABF material properties, Cu volume fraction, and edge clearance. The main findings can be summarized as follows.

1. Top-edge cracks near the RDL/glass interface pose the greatest risk compared to center-edge cracks. The risk of crack propagation increases linearly with the number of RDLs, with four-layer structures exceeding the fracture threshold.
2. Thinner glass panels only slightly increase the risk of crack propagation; however, at 100 µm thickness, the trend becomes pronounced, and the glass is highly vulnerable to cracking.
3. The choice of glass material has a pronounced impact on crack suppression. High-CTE glasses, including D263, Gorilla, and ceramic glass, effectively mitigate the risk of propagation.
4. Selecting appropriate ABF materials is also crucial for crack resistance. GZ-41 is a superior candidate for suppressing cracking due to their favorable combination of low CTE and moderate Young’s modulus.
5. Reducing edge clearance markedly increases the risk of edge crack propagation, whereas maintaining a clearance of at least 300 µm from Cu vias to the glass edge is essential for effective crack suppression.

Overall, this study establishes design-oriented insights that can guide the reliable integration of ultra-thin glass substrates in high-density packaging. Future work will extend these results to experimental validation and the development of industry-standard guidelines for large-area glass interposers.

## Figures and Tables

**Table 1:** Material properties used in the numerical analysis [9,26,29].

Materials	*E* (GPa)	*ν*	*α* (ppm/°C)	Stress-Free Temperature (°C)
Cu	91.7	0.34	17.6	110
ABF GX-92	5	0.3	39	180
Borofloat 33 glass	64	0.2	3.25	25

*E* is Young’s modulus, *ν* is Poisson’s ratio, *α* is coefficient of thermal expansion.

**Table 2:** Equivalent mechanical properties of RDL.

	*E_x_* (GPa)	*E_z_* (GPa)	*ν*_xy_	*ν*_xz_	G_xy_ (GPa)	G_xz_ (GPa)	*α*_xy_ (ppm/°C)	*α*_xz_ (ppm/°C)	Stress-Free Temperature (°C)
RDL	10.21	48.35	0.316	0.32	3.64	18.07	31.15	18.71	117

*E* is Young’s modulus, *ν* is Poisson’s ratio, *α* is coefficient of thermal expansion, G is shear modulus.

**Table 3:** Glass materials used in the numerical analysis [9,29,34,35].

Materials	*E* (GPa)	*ν*	α (ppm/°C)
Fused silica	73	0.16	0.57
Borofloat 33	64	0.2	3.25
D 263	72.9	0.21	7.2
Gorilla	71.5	0.21	8.14
Ceramic	67	0.29	9.3

*E* is Young’s modulus, *ν* is Poisson’s ratio, *α* is coefficient of thermal expansion.

**Table 4:** Four ABF materials used in the numerical analysis [26,27,28,29].

ABF Materials	*E* (GPa)	*ν*	*α* (ppm/°C)
GL-102	13	0.3	20
GX-92	5	0.3	39
GZ-22	6.4	0.3	31
GZ-41	9	0.3	20

*E* is Young’s modulus, *ν* is Poisson’s ratio, *α* is coefficient of thermal expansion.

**Table 5:** Equivalent material properties of RDL for different Cu percentage.

Cu Content (%)	*E_x_* (GPa)	*E_z_* (GPa)	*ν*_xy_	*ν*_xz_	G_xy_ (GPa)	G_xz_ (GPa)	*α*_xy_ (ppm/°C)	*α*_xz_ (ppm/°C)	Stress-Free Temperature (°C)
10	5.83	13.67	0.386	0.304	2.12	5.51	37.5	24.6	147
20	6.59	22.34	0.356	0.308	2.37	8.38	36.7	21.4	132
30	7.49	31.01	0.338	0.312	2.68	11.61	35.3	20.2	125
40	8.65	39.68	0.325	0.316	3.09	14.84	33.8	19.2	121
50	10.21	48.35	0.316	0.32	3.64	18.07	31.15	18.71	117
60	12.43	57.02	0.308	0.324	4.43	21.3	28.5	18.4	115
70	15.88	65.69	0.303	3.28	5.21	24.5	25.8	18.1	113
80	21.93	74.36	0.298	3.32	6.12	27.3	23.1	17.9	112
90	35.41	83.03	0.294	0.36	7.15	30.75	20.3	17.7	111

*E* is Young’s modulus, *ν* is Poisson’s ratio, *α* is coefficient of thermal expansion, G is shear modulus.

## Data Availability

The original contributions presented in this study are included in the article. Further inquiries can be directed to the corresponding author.
